# Supplementary material for: Qualitative classification of thrombus images as a way to improve quantitative analysis of thrombus formation in flow chamber assays
Source: PLoS One. 2024 Mar 11;19(3):e0299202. doi: 10.1371/journal.pone.0299202 (PMC10927075; doi:10.1371/journal.pone.0299202)
Supplement: S1 File — (DOCX) [file pone.0299202.s001.docx]

**Training of models in Ilastik**

1. Segmentation of aggregates irrespective of their morphology

As a first step a pixel classification module was used to annotate pixels. Input images were in .tiff format. All suggested features that Random Forest classifier can use for classification of pixels were selected in the feature selection applet. Two classes were created: pixels in the background and pixels in the area covered by platelets. The second class contained area which, according to the analyst, CD41-positive staining was observed. The outcome of this procedure were .h5 files consisting of probability maps where each pixel is assigned with a value that it belongs to a certain class. The trained model of pixel classification was saved as Ilastik “.ilp” file. As a next step an object classification module was used. This module uses two files as an input: an original .tiff file and a pixel probability map .h5 generated in a previous step. In a threshold applet a pixel class representing “platelet pixels” was selected as an input. All suggested features (excluding object location ) that Random Forest classifier can use for classification of objects were selected in the feature selection applet. One class of objects was created, i.e. all objects consisting of “platelet pixels” were classified as aggregates. The outcome of this procedure were 8-bit .tiff object prediction files where to each pixel being a part of aggregate a value of 255 was assigned while all the other pixels (background) had a value of 0. The trained model of object classification was saved as Ilastik “.ilp” file.

1. Segmentation of compacted aggregates

In pixel classification module pixels were assigned to two classes: a) background pixels, b) pixels forming CD41-positive structures. The outcome of this procedure were .h5 files consisting of probability maps where each pixel is assigned with a value that it belongs to a certain class. The trained model of pixel classification was saved as Ilastik “.ilp” file. As a next step an object classification module was used. This module used as an input two files: an original .tiff file and a pixel probability map .h5 generated in a previous step. In object classification module in a threshold applet a pixel class representing pixels classified as belonging to CD41-positive structures class were selected as an input. Two classes of objects were created. Objects with strong edge staining on most of the perimeter and with relatively darker centre were classified as compacted thrombi whereas objects where staining was more uniform, and the structures presumably less compacted, were classified to the second group. The outcome of the object classification in this case were two probability maps of objects in a form of a single 8-bit “.tiff” file. In one of them pixels forming each of the objects were assigned a value of probability that given object belongs to the actual compacted class whereas the other map contained values of probability that the object belonged to non-compacted dense class. To allow a better visualisation of the maps, the probability values were renormalized from 0-1 range to 0-255 range (a bit depth of an 8-bit image). The trained model of object classification was saved as Ilastik “.ilp” file.

**Quantification**

1. *Irrespective of morphology*

Images acquired in a single channel were collected in a folder named after the channel number, and the folders which consisted a single experiment were collected in one folder named after the experiment identifier (e.g. date).

When pipeline.py script is launched it prompts user to choose the folder which contains images to be analysed. The folder can contain subfolders. The script launches at first place Fiji macro (czi_to_tif.ijm) to convert .czi images to .tiff files. The original .czi files are replaced by .tiff files in the original folder. Once the operation is finished the script launches another python script (Ilastik_headless_pipeline.py) which runs Ilastik in headless mode. At first place pixel classification is performed on a given image and then object classification on the same image. The script performs the operation on all images contained in the main folder. Once the operation is finished the script launches Fiji macro (analyze_particles.ijm) which performs “analyze particles” operation to measure a series of parameters of each object in the image and generates .csv files containing these measured parameters for each image separately. After completion of this operation the python script (results_summary.py) is launched which generates .xlsx file. The file contains spreadsheets and each spreadsheet combines measured parameters for each image in a given subfolder.

1. *Only compacted thrombi*

This workflow was similar to the one described above with an exception of object classification by Ilastik and particle analyzis performed by FIJI.

As described in the “training” section above, the outcome of the object classification in this case were two probability maps in a form of a single “.tiff” file. In these maps pixels making up an object were tagged with probability values (renormalized to 0-255) that the object belongs to a respective class. In one of the maps it was a probability that an object belonged to the compacted aggregates and in the other that it belonged to the non-compacted aggregates. In order to obtain the object prediction map only for compacted aggregates, FIJI was used to split the tiff file and only the map presenting the probabilities for compacted aggregates was further analysed. To quantify the object properties the maps must have been thresholded with respect to the probability values so that only the relevant objects were taken into account. To this end the threshold value was set at a defined value so that only the objects which belonged to compacted aggregates class with the probability higher than this value were analyzed. Particle analysis and parameters calculated for each thrombus as well as their organization in excel file were the same as in the case of analyzis irrespective of morphology.

Pipeline.py script operation in this case is similar to this described in i) *Irrespective of morphology,* with two substantial differences:

1. The Ilastik models launched by Ilastik_headless_pipeline.py are different
2. Fiji macro ( area_thrombi.ijm) splits each object_probabilities.tif file to two files. The one which contains probability of compacted aggregates is thresholded at a value given in the script and particle analyses operation is performed on such thresholded maps.
